# EHMTI-0193. The impact of cognitive symptoms on migraine attack related disability

**DOI:** 10.1186/1129-2377-15-S1-D21

**Published:** 2014-09-18

**Authors:** R Gil-Gouveia, AG Oliveira, IP Martins

**Affiliations:** 1 Headache Center, Hospital da Luz, Lisboa, Portugal; 2 Pharmacy Department, Universidade Federal do Rio Grande do Norte, Rio Grande do Norte, Brazil; 3 Department of Clinical Neurosciences, Instituto de Medicina Molecular (IMM) Faculdade de Medicina Universidade de Lisboa, Lisboa, Portugal

## Introduction

The socio-economic impact of Migraine is related to work loss either by absenteeism or decreased work performance. Migraine associated cognitive dysfunction during an attack may be the cause of this difficulties.

## Aim

To analyze the presence and relevance of cognitive symptoms during migraine attacks, relating their intensity and symptom related disability with other migraine defining symptoms.

## Methods

Consecutive migraine patients of headache clinic fulfilled diaries scoring each migraine symptom (including cognitive symptoms) intensity and symptom related disability.

## Results

One hundred consecutive patients were included in this study (eight males, age average 31.2 ± 7.5 years), 34 (all females, age average 31.8 ± 8.8 years) returned information on 229 attacks, on average 6.7 per participant. This population had a moderate to severe impact of migraine (HIT-6 63.4 ± 4.4). Intensity of each symptom is always rated slightly higher than disability. Pain is the symptom scored with the highest intensity and disability, followed by cognitive symptoms (difficulty in thinking and worsening with mental effort) and photo and phonophobia.

## Conclusions

Cognitive symptoms are frequent during migraine attacks, their intensity and perceived symptom-related disability is second only to pain, during the attack. New acute migraine drugs trial should include cognitive evaluation as a secondary end-point, in order to be able to diminish decreased work performance and Migraine burden.

No conflict of interest.

